# Circulating Leukotriene B4 Identifies Respiratory Complications after Trauma

**DOI:** 10.1155/2012/536156

**Published:** 2012-03-07

**Authors:** Birgit Auner, Emanuel V. Geiger, Dirk Henrich, Mark Lehnert, Ingo Marzi, Borna Relja

**Affiliations:** Department of Trauma, Hand and Reconstructive Surgery, Hospital of the Goethe University Frankfurt am Main, 60590 Frankfurt, Germany

## Abstract

*Background*. Leukotriene B4 (LTB4), a proinflammatory lipid mediator correlates well with the acute phase of Acute Respiratory Distress Syndrome (ARDS). Therefore, LTB4-levels were investigated to determine whether they might be a useful clinical marker in predicting pulmonary complications (PC) in multiply traumatized patients. Methods: Plasma levels of LTB4 were determined in 100 patients on admission (ED) and for five consecutive days (daily). Twenty healthy volunteers served as control. LTB4-levels were measured by ELISA. Thirty patients developed PC (pneumonia, respiratory failure, acute lung injury (ALI), ARDS, pulmonary embolism) and 70 had no PC (ØPC). *Results*. LTB4-levels in the PC-group [127.8 pg/mL, IQR: 104–200pg/ml] were significantly higher compared to the ØPC-group on admission [95.6 pg/mL, IQR: 55–143 pg/mL] or control-group [58.4 pg/mL, IQR: 36–108 pg/mL]. LTB4 continuously declined to basal levels from day 1 to 5 without differences between the groups. The cutoff to predict PC was calculated at 109.6 pg/mL (72% specificity, 67% sensitivity). LTB4 was not influenced by overall or chest injury severity, age, gender or massive transfusion. Patients with PC received mechanical ventilation for a significantly longer period of time, and had prolonged intensive care unit and overall hospital stay. *Conclusion*. High LTB4-levels indicate risk for PC development in multiply traumatized patients.

## 1. Introduction

Trauma patients are at high risk of developing respiratory complications such as pneumonia, respiratory failure, Acute Lung Injury (ALI), Acute Respiratory Distress Syndrome (ARDS), and pulmonary embolism. Following multiple organ failure (MOF) and sepsis, respiratory complications are among the most common causes of morbidity and mortality for trauma patients surviving the initial postinjury phase [1–5]. The overall mortality from ARDS is still up to 50% [6–8]. Multiply traumatized patients have shown 10% mortality following ALI [9]. Approximately 20% of major trauma admissions develop ARDS or ARDS like pulmonary dysfunction. This represents one of the most frequent complications in these patients and is the major contributor to morbidity and mortality in trauma patients [3, 10, 11].

Several airway diseases including ALI/ARDS are closely associated with neutrophil infiltration of the airway wall [12]. Neutrophils release a variety of oxidants, as well as degradative and proteolytic enzymes, which induce lung inflammation with subsequent airway remodelling, microvascular damage, and lung tissue injury [12–14]. Persistence of neutrophils in the lungs is an important contributing factor to poor survival [15, 16].

Leukotriene (LT) B4 is a proinflammatory lipid mediator derived from the 5-lipoxygenase (5-LO) pathway of arachidonic acid metabolism [17–20]. LTB4 is a potent chemoattractant which also exerts leukocyte activating abilities and plays a crucial role in neutrophil migration [21–24]. LTB4 induces neutrophil adherence to endothelial cells, promotes chemotaxis, stimulates the generation and release of oxidants, and increases 5-LO activation in neutrophils, resulting in enhanced LTB4 synthesis [17, 18, 25]. Patients with pulmonary disease have elevated levels of LTB4 indicating its proinflammatory role [25–27]. LTB4 concentrations are enhanced in bronchoalveolar lage (BAL) fluid of ALI/ARDS and chronic obstructive pulmonary disease (COPD) patients [27, 28]. Recently, it has been reported that LTB4 and its metabolites, due to a “priming” effect on neutrophils, plays an important role in the development of polymorphonuclear-neutrophils-(PMN-) induced lung injury [29]. The priming effect of sequestered neutrophils in the lungs leads to their “hyperfunction.” This results in an exaggerated inflammatory cell response to a secondary stimulus potentially inducing lung complications [30–32]. Early identification of high-risk patients for respiratory complications after trauma is important in determining subsequent treatment. The potential prognostic role of LTB4 in major trauma patients, suffering lung complications in a later postinjury phase, remains unclarified.

We hypothesize that high levels of LTB4 in the plasma of multiply traumatized patients indicate not only a strong proinflammatory response, but may also serve to identify patients at risk for imminent lung complications.

## 2. Methods

### 2.1. Ethics

 This study was performed in the Goethe University Hospital with ethical approval (167/05, in accordance with the Declaration of Helsinki and following STROBE-guidelines) [33]. All patients signed the informed consent forms themselves or informed consent was obtained from relatives in accordance with ethical standards.

### 2.2. Patients

Inclusion criteria consisted of a history of acute blunt or penetrating trauma with an Injury Severity Score (ISS) ≥  16 in any patient between 18 and 80 years of age. Burns, concomitant acute myocardial infarction, and/or lethal injury were exclusion criteria.

Blood samples were obtained from 100 multiply traumatized patients on admittance to the emergency department (ED), and daily for 5 days following the trauma. Upon arrival at the ED, vital signs were documented. Trauma severity was scored using the Abbreviated Injury Scale (AIS) [34–36]. In addition, ISS was calculated [37]. Patients with an ISS from 16–24 were classified as substantially injured patients, patients with an ISS from 25–39 were substantially/severely injured patients and patients with an ISS ≥ 40 were considered severely injured patients.

Pulmonary complications were defined as nosocomial pneumonia, ALI/ARDS, pulmonary embolism, and/or respiratory failure as described below. Pneumonia was defined by radiologic, clinical, and bacteriologic findings with the presence of new pulmonary infiltrates and at least one of the following criteria: positive blood culture, BAL, and/or sputum culture [38]. Lung injury was assessed using the American-European Consensus Conference criteria for ARDS [39]. Pulmonary embolism was diagnosed by computed tomography (CT), and pulmonary edema was diagnosed either by CT scan or chest X-ray. Respiratory failure was defined as the need for prolonged weaning or reintubation.

 The control group included 20 healthy nonsmoking volunteers with un known chronic disease and no history of abdominal trauma or abdominal surgery within the past 24 months.

### 2.3. Blood Processing and Analysis

Blood samples were collected as early as possible after injury in prechilled ethylenediaminetetraacetic acid (EDTA) vacuum tubes (BD vacutainer, Becton Dickinson Diagnostics, Aalst, Belgium) and kept on ice. Blood was centrifuged at 2000  ×g for 15 minutes at 4°C. The supernatant was stored at −80°C until batch sample analysis.

The mean time between the injury and first blood sample taken directly upon admittance to the ED was 83 ± 7 min. Specimens were used for duplicate measurement of LTB4 levels. LTB4 was determined using a highly specific commercially available ELISA (LTB4 Parameter Assay Kit, R&D Systems, Minneapolis, USA) according to the manufacturer's instructions. The detection limit was 27.6 pg/mL for LTB4.

### 2.4. Statistics

Kolmogoroff-Smirnoff-Lillieford's test showed that the plasma concentration of LTB4 was not Gaussian-distributed. Median LTB4 levels for each of the 3 groups were compared using the Kruskal-Wallis test and the post hoc analysis was performed with Dunn's multiple comparison test. Data are presented as the median (interquartile range, IQR) or mean ± sem unless otherwise stated. A *P* value <0.05 was considered statistically significant. Receiver-operator curves were generated to analyze the optimal cutoff levels. GraphPad Prism 5.0 software (GraphPad Software Inc. San Diego, CA) was used to perform the statistical analysis and computations.

## 3. Results

The total group consisted of 100 patients (24 female, 76 male), 98% suffering from blunt and 2% from penetrating trauma. All patients were substantially injured (ISS: 34.0 ± 1.7). Of these, 30 patients with an ISS of 33.7 ± 1.6 developed secondary pulmonary complications. Seventy patients with an ISS of 34.1 ± 1.3 had no pulmonary complications. The AIS_chest_ was comparable in both groups (3.1 ± 0.2 in the ØPC group and 3.5 ± 0.2 in the PC group). Time on mechanical ventilation, length of stay in the ICU and hospital were significantly prolonged in the PC group. Additionally, more patients developed sepsis (*P* < 0.05), organ failure, and MOF in the PC group. In-hospital mortality was also increased.  Table 1 summarizes general patient characteristics and physiologic parameters in the study population.  Table 2 depicts the type, severity, and cause of injury.

**Figure 1:**
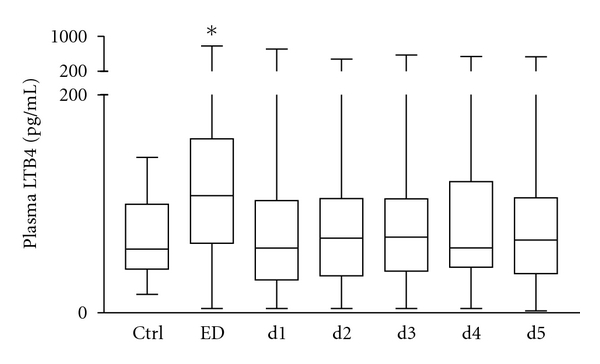
Summary of median (interquartile range) LTB4 (pg/mL) in multiply traumatized patients over a 5-day time course after admission (*n* = 100), and ctrl (*n* = 20). *P < 0.05 versus other groups.

Figure 1 shows the distribution of plasma LTB4 values in the first sample obtained in the ED and subsequent daily measurements for five consecutive days. Median concentrations (and IQR) of LTB4 in trauma patients on admission were significantly increased compared to healthy controls (106.1 (62–159) pg/mL versus 58.4 (36–108) pg/mL, *P* < 0.05, Figure 1). The LTB4 levels on admission were also significantly elevated compared with levels at day 1 until day 5.

**Figure 2:**
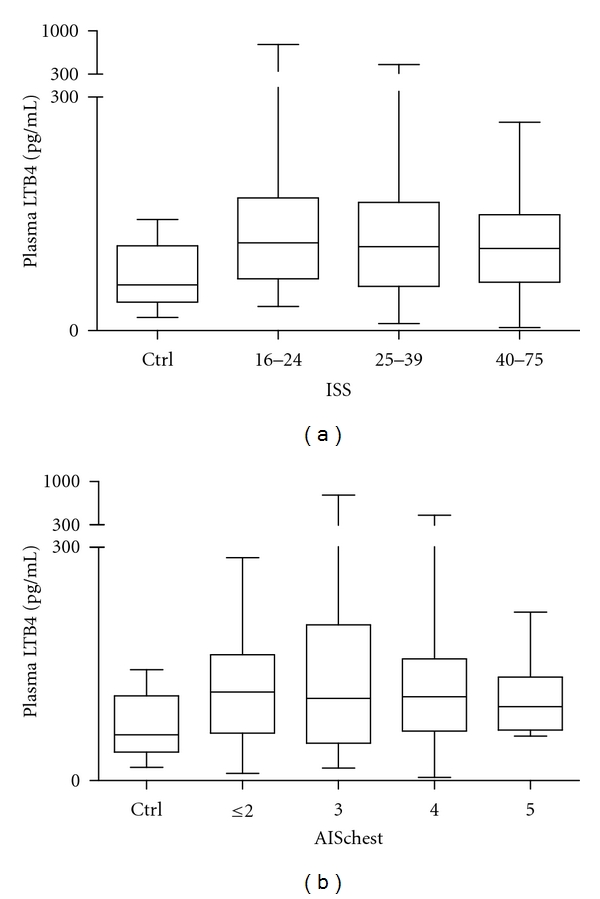
Summary of median (interquartile range) LTB4 (pg/mL) in ED samples in different groups of patients based on the overall injury severity (a) and the severity of chest injury (b). (a) ISS: 16–24, *n* = 17, ISS: 25–39, *n* = 54, ISS: ≥40, *n* = 29 and ctrl group, *n* = 10. (b) AIS_chest_ ≤ 2, *n* = 21, AIS_chest_ = 3, *n* = 23, AIS_chest_ = 4, *n* = 49, AIS_chest_ = 5, *n* = 7 and ctrl, *n* = 20.

 To investigate the relation between the injury severity and LTB4 concentrations determined in the ED, the study population was subdivided into three groups: seriously injured patients (ISS: 16–24, *n* = 17), seriously/severely injured patients (ISS: 25–39, *n* = 54), and severely injured patients (ISS: ≥40, *n* = 29). Plasma LTB4 concentrations in each group were markedly enhanced (112.8 (68–167) pg/mL, 107.6 (48–164) pg/mL, and 105.3 (62–148) pg/mL, resp.) compared with healthy volunteers 58.4 (36–108) pg/mL, but this tendency was not significant (Figure 2(a)).

The severity of chest trauma was assessed using the AIS chest scores. Patients without a relevant chest injury were graded as AIS_chest_ ≤ 2 (*n* = 21). Patients with serious and serious/severe chest injury (AIS_chest_ = 3 or 4) occurred most frequently (*n* = 23, and *n* = 49, resp.), whereas patients with an AIS_chest_ = 5 occurred less often (*n* = 7). Taken together, LTB4 levels were increased in all four trauma patient groups (AIS_chest_ ≤ 2: 113.9 (61–162), AIS_chest_ = 3: 105.8 (47–200), AIS_chest_ = 4: 107.7 (64–156), and AIS_chest_ = 5: 94.9 (65–133) pg/mL) compared with healthy volunteers, but this difference was not significant (Figure 2(b)).

**Figure 3:**
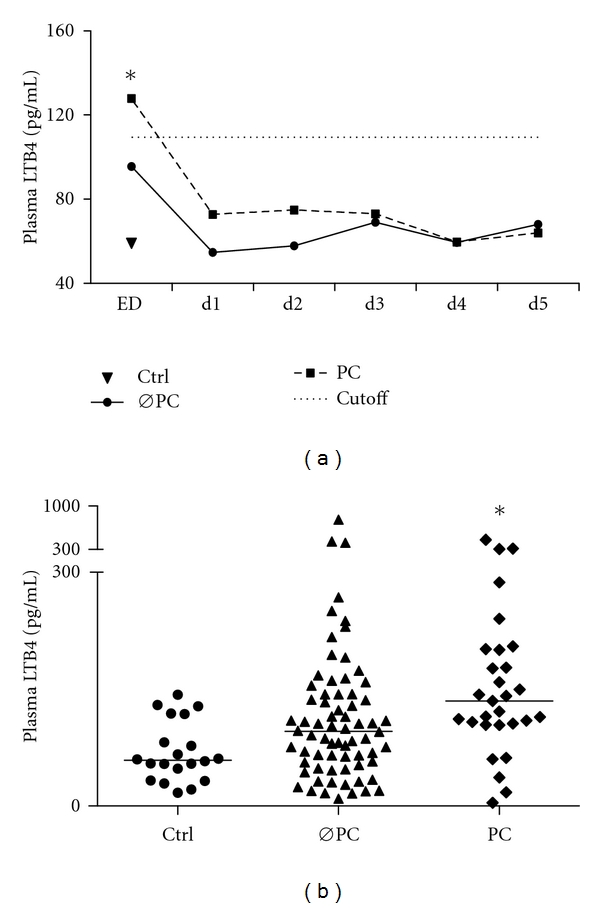
Summary of median LTB4 (pg/mL) in two patient groups based on the development of pulmonary complications (ØPC: no pulmonary complications, *n* = 70, and PC: pulmonary complications, *n* = 30) and ctrl, *n* = 20, *P<0.05 versus other groups: (a) time course in both groups, (b) LTB4 levels at the ED.

 However, comparing LTB4 levels taken in the ED of those patients who developed pulmonary complications (*n* = 30) following injury with those patients who did not develop pulmonary complications (*n* = 70) and healthy volunteers revealed a significant difference (127.8 (104–200) versus 95.6 (55–143) and 58.4 (36–108) pg/mL, resp., *P* < 0.05, Figures 3(a) and 3(b)). Follow-up LTB4 levels (day 1–day 5) showed that increased LTB4 levels in the ED in patients with pulmonary complications diminished in a time-dependent manner (Figure 3(a)). This effect was irrespective of the patients ISS or AIS_chest_ since subgroup analysis according to ISS or AIS_chest_ revealed no differences in LTB4 levels.

**Figure 4:**
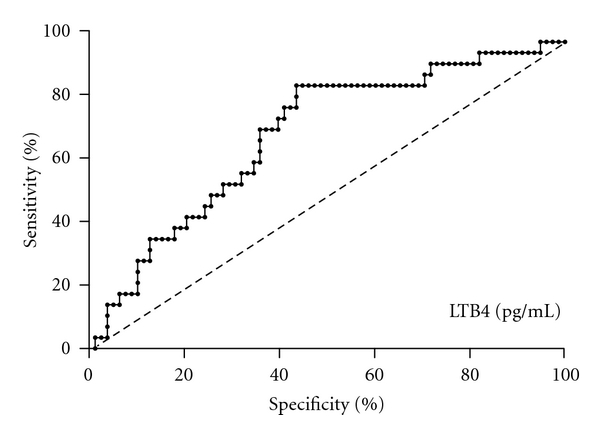
Receiver operating curve showing the optimal cutoff for LTB4 levels (109.6 pg/mL in predicting the presence or absence of postinjury pulmonary complications with 72% specificity and 67% sensitivity).

 Receiver operating characteristics (ROCs) analysis for LTB4 shows an optimal cutoff of 109.6 pg/mL with 72% specificity (95% CI: 0.61–0.81) and 67% sensitivity (95% CI: 0.49–0.84), for predicting pulmonary complications in a later posttraumatic course (Figure 4). The area under the ROC curve is 0.73.

Multiply traumatized patients with pulmonary complications needed significantly prolonged mechanical ventilation compared with patients without secondary pulmonary complications (18.4 ± 2.7 days versus 6.4 ± 0.8 days, resp., *P* < 0.05, Table 1). The mean ICU stay of all patients was 12.8 ± 1.3 days. Patients with pulmonary complications had a mean ICU stay of 22.4 ± 3.1 days versus 8.9 ± 0.9 days in patients without pulmonary complications (*P* < 0.05, Table 1). Patients with pulmonary complications also had significantly prolonged hospital stay compared with patients without pulmonary complications (32.5 ± 5.2 days versus 20.5 ± 1.7 days, resp., *P* < 0.05, Table 1). Sixteen patients developed sepsis, of those 69% had pulmonary complications (Table 1). Eight patients suffered from organ failure, and 4 patients had MOF, of those 63% and 75%, respectively, had pulmonary complications (Table 1). The mortality rate was enhanced for patients with pulmonary complications (10%) compared with patients without pulmonary complications (4%, Table 1).

## 4. Discussion

Respiratory complications, such as pneumonia, respiratory failure, ALI/ARDS, and pulmonary embolism are, next to MOF and sepsis, among the most common causes of late morbidity and mortality after trauma [1–5, 40]. An increased rate of pulmonary complication in severely injured trauma patients is closely associated with an excessive systemic and local inflammatory response including neutrophil influx [12, 40–42]. LTB4 represents a potent neutrophil chemoattractant and enhanced LTB4 levels are associated with pulmonary disease [25–28]. Despite the close association with airway disease, it remains unclear whether LTB4 is a reliable parameter for early identification of high-risk patients for pulmonary complications after multiple trauma.

This study shows that multiply traumatized patients with high LTB4 levels (cutoff at 109.6 pg/mL) in the initial phase are at high risk to develop posttraumatic pulmonary complications. The association of increased LTB4 in BAL fluid of patients with ALI/ARDS and COPD has previously been reported [27, 28]. The majority of clinical studies focused on the proinflammatory role of LTB4 in neutrophil infiltration and the subsequently induced lung injury in ICU patients [27–32]. The decisive role of neutrophils in several airway diseases, including ALI/ARDS, has been described [12–14]. Their persistence in the lungs is closely associated with poor survival [15, 16].

LTB4 is biosynthesized from arachidonic acid by the action of cytosolic phospholipase A_2_, 5-LO together with 5-LO-activating protein (FLAP) and leukotriene A4 hydrolase [43]. 5-LO activity is considered a key factor in LTB4 biosynthesis. LTB4 levels have been shown to correlate with tumor necrosis factor alpha levels and the number of neutrophils recovered from the BAL fluid of patients with ARDS [44]. Furthermore, LTB4 and its metabolites have been shown to cause increased neutrophil adherence to the pulmonary endothelial cell surface, reflecting neutrophil sequestration in the lung and the capillary bed and increasing vascular permeability [17, 29, 45, 46]. Inhibiting the 5-LO rate-limiting enzyme in LTB4 biosynthesis by intratracheal application of IL-8 has been shown to prevent lung injury and perfusate LTB4 increase in the lungs. Neutrophil chemotaxis *in vitro* was also inhibited [47]. Therefore, it might be concluded that the impact of enhanced systemic LTB4 concentrations in trauma patients presenting at the ED may reflect an ongoing systemic inflammation. This, in turn, may lead to the development of pulmonary complications. Therefore, the predictive relevance of LTB4 should be considered in this highly heterogeneous group of patients. Employing timely appropriate treatment (e.g., kinetic therapy, operative and ventilatory strategies) could thereby improve patient outcome.

Other factors have been reported to be associated with the development of posttraumatic pulmonary complications. The extent of chest trauma has been shown to increase respiratory complications, such as ALI/ARDS [48, 49]. Interestingly, we found no significant correlation between the degree of chest injury assessed by the AIS and the rate of posttraumatic pulmonary complications. This may be due to the study having been conducted at a single clinic with a limited number of patients (*n* = 100). In a multivariate statistical analysis, we found that the effect of trauma severity on LTB4 levels as well as the development of pulmonary complications is considered not significant (data not shown). Evaluation of data from patients with hypoxemic respiratory failure (*n* = 8) has shown that LTB4 levels (at ED) were significantly enhanced in this group compared to healthy volunteers, but did not differ markedly from 22 patients with nonhypoxemic respiratory complication (data not shown). In the present study, the mortality was 6% in a cohort of trauma patients with considerable injury (mean ISS > 33). Blunt trauma as the major type of injury in over 90% of patients was in accordance with other European studies [50, 51]. Dysregulated immune response after trauma has been suggested to contribute to complications, such as sepsis and MOF. The incidence of sepsis and MOF vary strongly in the literature [2, 52–56]. In the present study, sepsis occurred in 16% and MOF in only 4% of patients. In line with the literature, trauma patients with pulmonary complications constitute the majority of patients who develop sepsis and/or MOF [52]. Interestingly, in the present study, patients developing pulmonary complications were not more severely injured than patients without pulmonary complications, as had been expected from previous reports [52]. The clinical course was strongly affected by the presence of respiratory complication with a prolonged ICU stay, days on mechanical ventilation, and longer hospital stay.

Pulmonary complications after severe trauma markedly affect the clinical course. The predisposing factors for these patients at risk are not fully understood and their identification before clinical manifestation of complications remains a challenge. Most clinical scoring systems have been developed to compare populations, while their predictive power is limited. The lung organ failure scoring (LOFS) method has been developed to estimate the risk for pulmonary complications in trauma patients [57]. Its effectiveness still needs to be assessed in prospective clinical studies.

In conclusion, to stratify the risk for later pulmonary complications the results presented here encourage LTB4 assessment early after trauma. In the present study, the LTB4 AUC is quite small. Enhanced patient numbers, especially in the group of patients with pulmonary complications, could strengthen the hypothesis that LTB4 may be of predictive value. While the pathophysiological sequelae of increased LTB4 release is principally understood, identification of relevant effects such as neutrophil adherence or edema formation need demonstration in the clinical setting. Therefore, clinical studies with larger numbers of patients are required to clarify the role of LTB4 in pulmonary complications and resolve its predictive efficacy.

## Figures and Tables

**Table 1:** Summary of patient characteristics and physiologic parameters in the investigated groups (ØPC: no pulmonary complications, PC: pulmonary complications and ctrl, data are presented as mean ± SEM unless otherwise stated).

Patient characteristics	All patients (*n* = 100)	PC (*n* = 30)	ØPC (*n* = 70)	ctrl (*n* = 20)	*P* value(PC versus ØPC)
Age (years)	39.2 ± 1.7	42.8 ± 3.4	37.9 ± 1.9	32.3 ± 2.8	0.2241
Sex (male)	76	20	56	7	—
Injury severity score	34.0 ± 1.0	33.7 ± 1.6	34.1 ± 1.3	—	1.0000
Surgery after admission	69.0%	76.7%	70.8%	—	0.6218
Thoracic drainage	34.0%	53.3%	25.7%	—	0.0191
Packed red blood cells (pRBC)/250 mL (24 h)	7.4 ± 1.2	8.6 ± 1.9	7.2 ± 1.5	—	0.2739
Massive transfusion (≥10 units pRBC in 24)	20.0%	26.7%	17.4%	—	0.6007
Haemoglobin (g/dL)	11.5 ± 0.3	9.9 ± 0.7	12.6 ± 0.3	—	0.0044
Intubation	73.0%	96.7%	81.4%	—	0.0762
Intubation duration (*d*)	9.9 ± 1.1	18.4 ± 2.7	6.4 ± 0.8	—	<0.0001
Rotational bed therapy	62.0%	86.7%	51.4%	—	0.0020
Rotational bed Therapy (*d*)	3.9 ± 0.5	5.3 ± 0.7	3.4 ± 0.6	—	0.0070
Intensive care period (*d*)	12.8 ± 1.3	22.4 ± 3.1	8.9 ± 0.9	—	<0.0001
Hospital stay	24.1 ± 2.0	32.5 ± 5.2	20.5 ± 1.7	—	<0.0001
Sepsis (*n*/%)	16/16.0%	11/36.7%	5/7.1%	—	0.0162
Organ failure (*n*/%)	8/8.0%	5/16.7%	3/4.3%	—	0.3137
MOF (*n*/%)	4/4.0%	3/10.0%	1/1.4%	—	0.4889
Hospital mortality (*n*/%)	6/6.0%	3/10.0%	3/4.3%	—	0.6390
LTB4 (pg/mL, interquartile range)	105.9 (96.7)	127.8 (96.5)	95.6 (88)	58.4 (72.1)	0.0140

**Table 2:** Overview of type and mechanisms of injuries within the two investigated groups groups (ØPC: no pulmonary complications, PC: pulmonary complications and ctrl, data are presented as mean ± SEM).

Group	All patients (*n* = 100)	PC (*n* = 30)	ØPC (*n* = 70)	*P* value (PC versus ØPC)
Abbreviated injury severity-scale (AIS, mean ± sem)				
AIS head	2.5 ± 0.2	2.3 ± 0.3	2.6 ± 0.2	0.4332
AIS chest	3.2 ± 0.1	3.5 ± 0.2	3.1 ± 0.2	0.1008
AIS abdomen	1.9 ± 0.1	1.9 ± 0.2	2.0 ± 0.2	0.8208
AIS extremities & external	2.2 ± 0.1	2.4 ± 0.2	2.2 ± 0.2	0.3601

Mechanism of Injury (*n*)				
Blunt	98	29	69	—
Penetrating	2	1	1	—

Accident mechanism (*n*/%)				
Road accident	69/69.0%	21/70.0%	48/68.6%	
Motorcycle	30/30.0%	4/13.3	26/37.1%	
Car	28/28.0%	12/40.0%	16/22.9%	
Pedestrian	6/6.0%	4/13.3%	2/2.9%	
Bicyclist	5/5.0%	1/3.3%	4/5.7%	
Fall	24/24.0%	6/20.0%	18/25.7%	
>3 m	20/20.0%	6/20.0%	14/20.0%	
<3 m	4/4.0%	—	4/5.7%	
Other	7/7.0%	3/10.0%	4/5.7%	
